# Leiomyoma of the nasal cavity: case report

**DOI:** 10.1016/S1808-8694(15)31185-X

**Published:** 2015-10-19

**Authors:** Pedro Paulo Vivacqua da Cunha Cintra, Wilma Terezinha Anselmo Lima, Aguilar Rodrigues Junior

**Affiliations:** 1 M.S. in otolaryngology - FMUSP - Ribeirão Preto, Assistant Physician - Núcleo de Otorrinolaringologia de São Paulo; 2 Associate Professor - Department of Otolaryngology and Ophthalmology and Head and Neck Surgery - HCFMRP-USP; 3 MD. Otolaryngologist - Assistant Physician - Núcleo de Otorrinolaringologia de São Paulo

**Keywords:** leiomyoma of the nasal cavity, paranasal sinus neoplasms

## CASE

M.AH, female, 54 years, born and raised in São Paulo, with repetition epistaxis, nasal obstruction and hyposmia for two years. A solid tumor was found in her nose, coming from the middle meatus and partially obstructing her left nasal cavity.

We performed a contrasted CT scan of her paranasal sinuses and a nasofibroscopy with biopsy. The material was then sent for a pathology exam and immunohistochemistry, which confirmed the diagnosis of a mesenchymal neoplasia originating from the smooth muscle.

Surgery was performed under endoscopic view, with a 30° 4mm scope, when we resected the posterior portion of the left middle concha. The patient remained without nasal packing in the postoperative period, and did not have complications.

### Anatomical and Histopathological Findings

A solid brownish tumor, measuring 4.0 × 2.0 × 2.0cm of regular borders.

Under light microscopy we noticed proliferation of elongated cells of low histological aggressiveness, portraying a phenotype suggesting leiomyoma. Histology cross-sections showed a proliferation of smooth muscle fibers, without atypia, in longitudinal and cross-sectional bundles, separated by a mild fibro-conjunctive stroma, vascularized, covered by respiratory mucosa modified by unspecific inflammatory alterations.

### Immunohistochemistry

We used the strepto-avidine-biotine complex method /HRP, investigating antigens Vimentin, PS100, Actine ML, CD34 and Enolase.

## RESULTS

Positive ML Vimentin and Actine, PS 100, negative CD34 and negative Enolase.

The histological pattern profile corresponds to that of a mesenchymal neoplasia originating from the smooth muscle.

## DISCUSSION

Leiomyomas are rare in the nasal cavity and in paranasal sinuses because there is very little smooth muscle in the nose, it is only present in the middle layer of blood vessels and in hair erection muscles. The origin is uncertain, it is likely that they should reach the smooth muscle on the vessel wall, the hair erection muscles or aberrant undifferentiated mesenchima.^2^

Smooth muscle tumors can be considered malignant even when the rate of mitoses is extremely low.^2^

High differentiation of tumor cells, minimum pleomorphism and the low degree of mitotic activity indicate that this is a benign tumor.^3^

Josephson et al.^4^ studied six cases of leiomyosarcoma present within benign tumors. This indicates the possibility of a benign tumor becoming a leiomyosarcoma.

In the few reported cases of leiomyoma of the nasal cavity and paranasal sinuses, according to the literature review done by Van Ingen^5^, the patients had between 42 and 76 years of age. There is a case of leiomyoma reported by Papavasilau6, in a five year old girl, which in fact was a leiomyoma variant, a leiomyoblastoma, which is a tumor that stems from remaining embryonic tissue.

There is a certain predilection for females in a 3:1 ratio, with mean age of 50 years for women and 60 years for men.

Less than 1% of leiomyomas happen in the head and neck.^3^ Nose and paranasal sinuses leiomyoma cause nasal obstruction (due to mass expansion), facial pain and headache (caused by obstruction of the mucociliary movement and that of the paranasal sinuses' drainage ostia), acute sinusitis and epistaxis.

Llorente^7^ classifies leiomyomas in 3 types: vascular, non-vascular, leiomyoblastoma or epithelioid. Our case was of the vascular type.

## COMMENTS

Although rare, leiomyomas must be kept in mind for the differential diagnosis of benign intranasal tumors.
